# Transcriptome-based gene profiling provides novel insights into the characteristics of radish root response to Cr stress with next-generation sequencing

**DOI:** 10.3389/fpls.2015.00202

**Published:** 2015-03-31

**Authors:** Yang Xie, Shan Ye, Yan Wang, Liang Xu, Xianwen Zhu, Jinlan Yang, Haiyang Feng, Rugang Yu, Benard Karanja, Yiqin Gong, Liwang Liu

**Affiliations:** ^1^National Key Laboratory of Crop Genetics and Germplasm Enhancement, College of Horticulture, Nanjing Agricultural UniversityNanjing, China; ^2^Department of Plant Sciences, North Dakota State UniversityFargo, ND, USA; ^3^Zhengzhou Vegetable Research InstituteZhengzhou, China

**Keywords:** radish, transcriptome, Solexa sequencing, Cr stress, DEGs, RT-qRCR

## Abstract

Radish (*Raphanus sativus* L.) is an important worldwide root vegetable crop with high nutrient values and is adversely affected by non-essential heavy metals including chromium (Cr). Little is known about the molecular mechanism underlying Cr stress response in radish. In this study, RNA-Seq technique was employed to identify differentially expressed genes (DEGs) under Cr stress. Based on *de novo* transcriptome assembly, there were 30,676 unigenes representing 60,881 transcripts isolated from radish root under Cr stress. Differential gene analysis revealed that 2985 uingenes were significantly differentially expressed between Cr-free (CK) and Cr-treated (Cr600) libraries, among which 1424 were up-regulated and 1561 down-regulated. Gene ontology (GO) analysis revealed that these DEGs were mainly involved in primary metabolic process, response to abiotic stimulus, cellular metabolic process and small molecule metabolic process. Kyoto encyclopedia of genes and genomes (KEGG) enrichment analysis showed that the DEGs were mainly involved in protein processing in endoplasmic reticulum, starch and sucrose metabolism, amino acid metabolism, glutathione metabolism, drug and xenobiotics by cytochrome P450 metabolism. RT-qPCR analysis showed that the expression patterns of 12 randomly selected DEGs were highly accordant with the results from RNA-seq. Furthermore, many candidate genes including signaling protein kinases, transcription factors and metal transporters, chelate compound biosynthesis and antioxidant system, were involved in defense and detoxification mechanisms of Cr stress response regulatory networks. These results would provide novel insight into molecular mechanism underlying plant responsiveness to Cr stress and facilitate further genetic manipulation on Cr uptake and accumulation in radish.

## Introduction

Heavy metal (HM) contamination has become one of the major worldwide environmental problems affecting soil, water and human health, leading to a considerable reduction in yield and food safety (Chandra and Kulshreshtha, 2004). An elevated content of HMs in human diet constitutes a potential health risk through food chain, vegetable crops, representing an important pathway for the movement of potentially toxic metals from soil to human beings (Gupta et al., 2012). Chromium (Cr), the seventh most abundant metal on earth, is one of the most toxic HMs to living organisms and ecosystems (Katz and Salem, 1994). Cr resource is mainly from industrial process products such as leather tanning, electroplating, steel production, metal finishing, catalyst application, pigment manufacturing and metal corrosion inhibitors (Shanker et al., 2009). Trivalent Cr (III) and hexavalent Cr (VI) species are the stable forms of Cr exist in the earth's crust. Accumulation of Cr in plants can interfere with several metabolic processes such as photosynthesis, water relation and uptake of nutrients. These stresses could reduce root growth and phytomass, induce chlorosis in plants, wilting and plasmolysis in root cells and finally plant death (Hayat et al., 2011). Being a strong oxidizer, Cr (VI) at even low concentration is highly toxic and more mobile in soil/water systems, and is also known to cause allergies, irritations, and even respiratory track disorders to human beings (Shanker et al., 2005).

In recent years, the next-generation sequencing (NGS) platform including Illumina/Solexa sequencing technology has provided a powerful tool for RNA sequencing, transcriptome assembly and gene expression profiling (Mochida and Shinozaki, 2011). NGS-based RNA-Seq is a very powerful technology for transcriptomics studies, and enables us to investigate the gene activities of organisms in diverse tissues and different stages under various conditions (Geng et al., 2011). Based on genome-wide expression profiles by high throughput sequencing, digital gene expression (DGE) could be employed to characterize, identify and quantify rare gene transcripts on the global transcriptome level without prior sequence knowledge (Strickler et al., 2012). In recent years, RNA-seq has provided a useful tool for identification of related genes and their expression patterns in crop pests and plant species responding to various biotic and abiotic stresses (Nachappa et al., 2012; Yu et al., 2012). High-throughput Solexa/Illumina sequencing was successfully applied to characterize gene expression of soybean under salt, saline-alkali and drought stress (Fan et al., 2013). In order to investigate head smut disease-resistance mechanisms, DGE analysis was used to identify the transcriptional changes in the roots of maize (Zhang et al., 2013). Many reports showed that the DGE approach has provided valuable tools for qualitative and quantitative gene expression analysis (Yamaguchi et al., 2010; Li et al., 2014).

Cr is absorbed by plant mainly through the roots. With transcriptomic and metabolomic techniques, some vital genes encoding PDR-like ABC transporter, multidrug resistance protein 4 and glutathione S-transferase GSTU6 were identified to be involved in response to Cr stress in rice (Dubey et al., 2010). Even though the molecular mechanisms of Cr stress effect on plants have been under scrutiny for decades, critical genes involved in response to Cr stress were not well identified and characterized in root vegetable crops. Radish (*Raphanus sativus* L., 2n = 2x = 18), belonging to the Brassicaceae family, is a major annual or biennial worldwide root vegetable crop especially in East Asia. However, most reports on Cr in plants have been concerned with its effects on plant growth, uptake, toxicity, translocation, and soil-plant relationships (Panda and Choudhury, 2005; Shanker et al., 2005). A comprehensive investigation on the molecular mechanism underlying Cr absorption and transport is urgently required in radish.

So far, there is no report on systematic identification and characterization of critical genes involved in Cr- responsiveness, uptake, transportation and accumulation in radish. Moreover, it is important to identify the key genes and clarify the molecular mechanism of Cr stress-response for altering the accumulation of Cr in radish plant through genetic improvements. In this study, RNA-Seq approach was employed with genome-wide transcriptional profiling of the without (control) and 600 mg/L K_2_Cr_2_O_7_ treatment to identify differentially expressed genes (DEGs) under Cr stress in radish roots. The aim of this study was to identify key DEGs under Cr stress in radish roots. With the high-throughput sequencing technique applied, a large number of unigenes, Cr responsive DEGs and their expression patterns were successfully obtained, and the expression profiling of a proportion of differentially regulated genes were validated by RT-qPCR. Furthermore, based on DEGs enrichment in the corresponding pathway, a hypothetical model of Cr stress-response regulatory network in radish was proposed. This study represents a first comprehensive transcriptome-based characterization of Cr responsive DEGs in radish roots, these results would provide fundamental insights into the complex Cr-responsive gene regulatory networks, and facilitate further studies on molecular genetic mechanisms underlying plant responses to Cr stress in root vegetable crops.

## Materials and methods

### Plant material

A high-Cr-accumulation radish advanced inbred line, “NAU-RG06,” was used in the study. Germinated seeds were sown in plastic pots with matrix and soil at a ratio of 1:1 and cultivated in a growth chamber at 25°C day/18°C night with a 14 h light/10 h dark photoperiod. Seedlings of 30 days old were transferred into liquid medium in a plastic box and grown for 3 days. Cr treatment was carried out under the same growth conditions by soaking the root in a K_2_Cr_2_O_7_ (Cr^6+^) solution of 0 (CK) and 600 mg L^−1^ (Cr600) for 72 h. After treatments, at least three replicates of roots for each treatment were harvested and immediately frozen in liquid nitrogen for further use.

### RNA isolation and illumina sequencing

Total RNA was extracted from root samples using TRIzol reagents (Tiangen Biotech Co., Ltd., China) according to the manufacturer's instructions. The RNAs were treated with RNase-free DNase I to eliminate contaminated genomic DNA. Two radish cDNA libraries, CK and Cr600, were constructed from control and 600 mg L^−1^ K_2_Cr_2_O_7_ treated root samples using the Illumina Paired End Sample Prep Kit. Briefly, poly (A) mRNA was enriched from total RNA using Sera-mag Magnetic Oligo (dT) Beads (Thermo Fisher Scientific, USA) and then mRNA-enriched RNAs were chemically fragmented to short pieces using the fragmentation solution (Ambion, USA). Double-stranded cDNA was generated using the Superscript Double-Stranded cDNA Synthesis Kit (Invitrogen, USA). After that, the Illumina Paired End Sample Prep kit was used for RNA-seq library construction and was then sequenced using Illumina HiSeq™ 2000.

### *De novo* transcriptome assembly and annotation

Illumina pipeline was used for filtering the raw sequence reads. The 3′ adaptor sequence was removed from raw sequences. All low-quality tags, such as short tags (<25 nt), empty tags, and tags with only one copy number were removed. *De novo* transcriptome assembly was accomplished from all the clean reads with the Trinity program and unigenes were generated (Grabherr et al., 2011; Wang et al., 2013). Only sequences with perfect homology or not more than two nucleotide mismatches were considered for conservative and accurate annotation. The assembled transcripts were further used as query sequences to search against NCBI non-redundant (nr) protein and COG (Clusters of orthologous groups of proteins) databases with BLASTX. For nr annotation, the Blast2GO program was used to get GO (Gene Ontology) annotation of assembled transcripts (Conesa et al., 2005). After getting GO annotation for assembled transcripts, WEGO software was employed to fulfill GO functional classification (Ye et al., 2006).

### Identification of differentially expressed genes

The clean reads were mapped to the reference database which included whole radish ESTs and unigenes from NCBI and transcriptome sequences using SOAPaligner/soap2 (Li et al., 2009). RPKM (reads per kilobase of exon model per million mapped reads) were used to evaluate the gene expression value and quantify transcript levels (Mortazavi et al., 2008). Using the DEGseq program, significantly differential gene expression was identified between the CK and Cr600 libraries (Wang et al., 2010). The false discovery rate (FDR) was used to determine the threshold *p* value in multiple tests procedure described by Benjamini and Yekutieli (2001). In this study, a stringent of FDR ≤ 0.001 and log_2_ | FC (ratio of stress/control) | ≥2 was used as the threshold to judge the significant difference of gene expression.

### Go and KEGG pathway enrichment analysis of DEGs

Functional classes were assigned according to GO mapping provided by the ensemble database. KEGG (Kyoto encyclopedia of genes and genomes) pathway analysis was based on the comparative results between our mapping genes and the current KEGG database (Kanehisa et al., 2008). The differentially expressed genes (DEGs) were used for GO and pathway enrichment analysis, and a corrected *p* ≤ 0.05 was selected as a threshold level of significance to determine enrichment in the gene sets (Wang et al., 2013).

### RT-qPCR analysis

Quantitative RT-PCR analysis was used to validate the expression of the candidate genes. RT-qPCR was conducted on MyiQReal-Time PCR Detection System (Bio-Rad) using a SYBR Primix Ex Taq kit (TaKaRa, Dalian, China) according to the manufacturer's instructions. Gene-specific primers and a *β-actin*-specific primer pair were designed according to the gene sequences using Beacon Designer 7.0 software. Each reaction was prepared in a total volume of 20 μl containing 10 μl SYBR Green mix, 2 μl diluted cDNA and 0.2 μM of each primer. Amplification was carried out with the following cycling parameters: heating for 10 s at 95°C, 40 cycles of denaturation at 95°C for 5 s, annealing at 58°C for 30 s and extension at 72°C for 10 s (Xu et al., 2012). Each sample was analyzed in triplicates and the expression values were normalized against β−actin. The molecular weight of the products was confirmed via diagnostic agarose gel and the melting curves were analyzed. Analysis of the relative gene expression data was conducted using the 2^−ΔΔ*C*_T_^ method (Livak and Schmittgen, 2001).

## Results

### Radish root transcriptome assembly and transcript function classification

**Figure 1:**
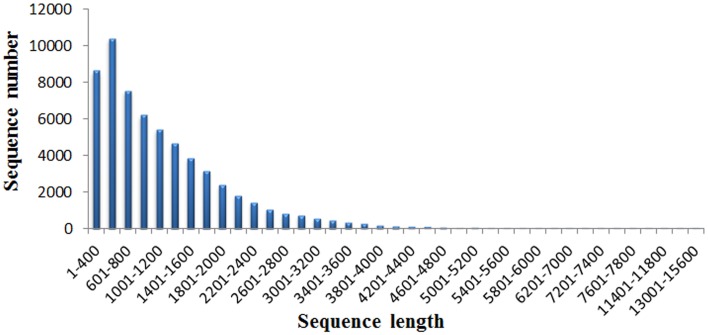
**The length distribution of the assembled transcripts**.

A total of 37.95M and 27.45M raw reads were generated from CK and Cr600 library with Illumina Solexa sequencing technology, respectively. Before mapping these sequencing reads to the reference genome, the reads with connectors, filtered low-length reads and low-quality reads were removed. A total of 26.38M (69.52%) and 15.03M (54.74%) 101-nt clean reads were generated in cDNA libraries of CK and Cr600, respectively (Table 1). The sequencing reads were aligned against the combined radish reference database, and a total of 60,881 isogenes or 30,676 unigenes with 71,526,014 nt were filtered out for the differential expression analysis. Moreover, the average length of the isogene was 1,174.85 bp, and the largest and shortest isogene was 15,585 and 306 bp, respectively. The sequence length of these assembled transcripts was mainly distributed between 306 and 3000 bp with 401 and 600 bp length in the highest abundance (Figure 1).

**Table 1:** **Reads from RNA-Seq library sequencing**.

	**CK**		**Cr600**	
	**Read number**	**Percent (%)**	**Read number**	**Percent (%)**
Total reads	37,948,330	100	27,451,966	100
Connector reads	7444	0.02	2,799,028	10.20
Low-length and low-quality reads	11,559,006	30.46	12,227,330	44.54
Clean reads	26,381,880	69.52	15,026,358	54.74

**Figure 2:**
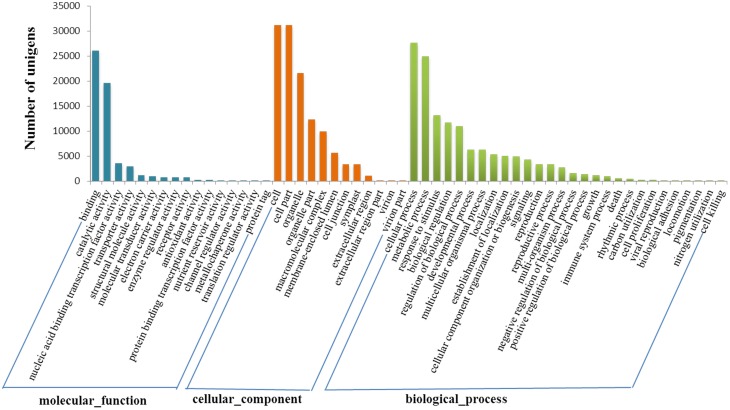
**Gene ontology classification of assembled transcripts**.

Gene ontology (GO) analysis indicated all these genes were distributed in three categories, namely biological process, cellular component and molecular function. Totally 42,178 transcript sequences (69.28%) were assigned to 57 GO terms at the second level. Cellular process (27,602 sequences, 65.44%), metabolic process (24,851 sequences, 58.92%) and response to stimulus (13,134 sequences, 31.14%) accounted for the main part in biological process category. In relation to cellular component, there were sequences associated with cell part (31,102 sequences, 73.74%) and organelle part (12,247 sequences, 29.04%) which represented the most abundant categories. The sequences associated with binding (26,066 sequences, 61.80%) and catalytic activity (19,614 sequences, 46.50%) were dominant in the molecular function category (Figure 2).

**Figure 3:**
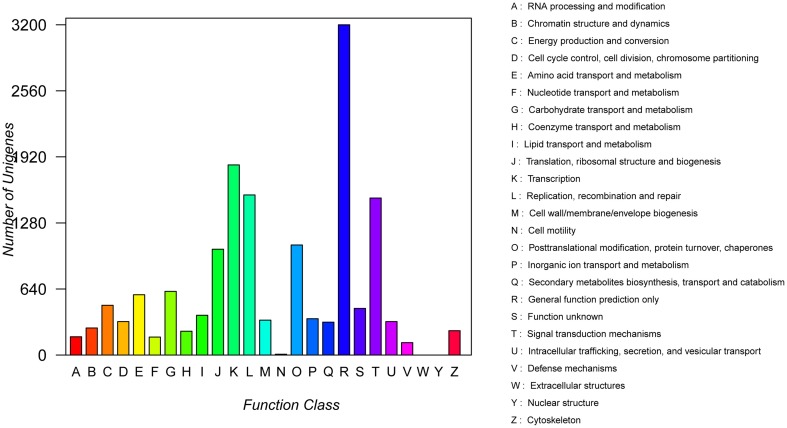
**COG function classification of unigenes**.

COG analysis showed that a total of 15,590 transcript sequences with high homology were grouped into 23 functional categories (Figure 3). The first three largest categories were “general function prediction only” (3200, 20.5%), “transcription” (1842, 11.8%), “replication, recombination and repair” (1551, 9.95%), followed by “signal transduction mechanisms” (1521, 9.8%) and “post translational modification, protein turnover, chaperones” (1066, 6.8%) (Figure 3; Table S1).

### Identification and functional analysis of DEGs under Cr stress

**Figure 4:**
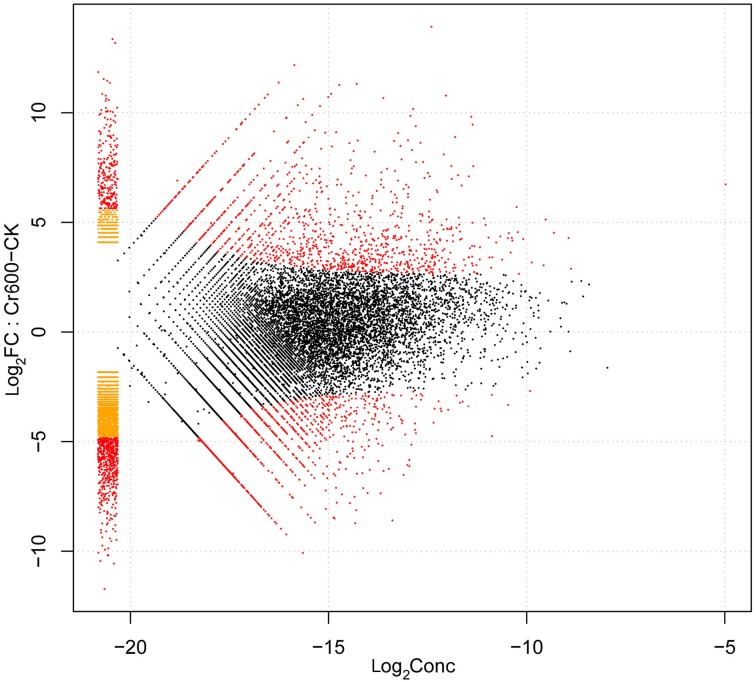
**Volcano plot of gene expression difference between Cr600 and CK libraries**.

By comparing two Solexa libraries (CK and Cr600), a great number of differentially expressed reads were identified. To study a subset of genes that were associated with Cr stress, we analyzed the most differentially regulated tags with a log_2_ratio ≥ 2 or ≤ −2, FDR ≥ 0.001. Using these standards, 6295 isogenes (2985 unigenes) were identified to be differentially expressed. Among all the differentially expressed genes (DEGs), 2842 isogenes (1424 unigenes) were identified to be up-regulated and the rest were down-regulated (Figure 4).

GO analysis was performed to predict functions by mapping each DEGs into the records of the GO database. According to Bonferroni-corrected *P* ≤ 0.05, a total of 214 terms for the up-regulated transcripts were enriched, and 269 terms were down-regulated. In the GO cellular component class, there were more up-regulated genes annotated for cell part (GO: 0044464), intracellular part (GO: 0044424) and cytoplasmic part (GO: 0044444). While the down-regulated genes were mainly annotated with membrane (GO: 0016020), plasma membrane (GO: 0005886) and intrinsic to membrane (GO: 0031224) (Tables S2, S3). In the GO molecular function class, many up-regulated genes were annotated by catalytic activity (GO: 0003824), oxidoreductase activity (GO: 0016491) and transporter activity (GO: 0005215). The down-regulated genes were mainly annotated by binding (GO: 0005488) and ATP binding (GO: 0005524) (Tables S2, S3). In the GO biological process class, there was a higher number of DEGs annotated by response to abiotic stimulus (GO: 0009628), primary metabolic process (GO: 0044238), cellular metabolic process (GO: 0044237) and small molecule metabolic process (GO: 0044281) (Tables S2, S3).

**Figure 5:**
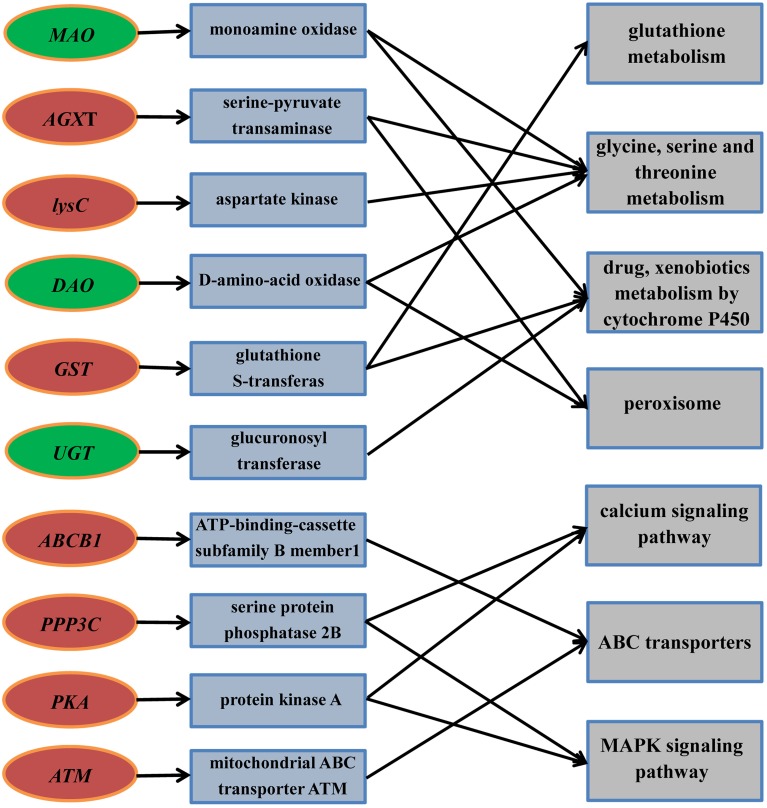
**The key candidate genes and their interaction with major pathways under Cr stress of radish roots**. Red and green boxes represent up-and down-regulated candidate genes, respectively. Blue and gray boxes represent gene definition and major pathways, respectively.

With Pathway-based analysis specific biological functions could be assigned to genes (Figure 5). A total of 3259 DEGs were annotated by KEGG pathway analysis, and a total of 11 and 15 significantly enriched pathways were found for up- and down-regulated DEGs, respectively (Tables 2, 3). For the up-regulated isogenes, the primarily enriched pathways included protein processing in endoplasmic reticulum [ko04141], glutathione metabolism [ko00480], drug metabolism - cytochrome P450 [ko00982] and xenobiotics metabolism by cytochrome P450 [ko00980] (Table 2; Figure 5). Down-regulated genes were mainly involved in starch and sucrose metabolism [ko00500], amino sugar and nucleotide sugar metabolism [ko00520] and pentose and glucuronate interconversions [ko00040] (Table 3). From the result of pathway enrichment analysis, it could be suggested that some genes might interact with each other to play specific roles in certain biological processes (Figure 5).

**Table 2:** **The significantly enriched pathways of up-regulated DEGs**.

**Pathway**	**Id**	**Sample number**	**Background number**	***P*-value**	**Corrected *P*-value**
Drug metabolism—cytochrome P450	ko00982	21	70	2.29E-09	7.37E-07
Metabolism of xenobiotics by cytochrome P450	ko00980	19	67	3.65E-08	5.88E–06
Protein processing in endoplasmic reticulum	ko04141	61	472	3.58E-07	2.88E-05
Glutathione metabolism	ko00480	25	134	1.99E-06	0.000106593
Cyanoamino acid metabolism	ko00460	15	74	8.03E-05	0.002872626
Alpha-Linolenic acid metabolism	ko00592	12	55	0.00019641	0.006324488
Glucosinolate biosynthesis	ko00966	8	28	0.00032714	0.008778165
Glycine, serine and threonine metabolism	ko00260	19	127	0.0006472	0.015116907
Tropane, piperidine, and pyridine alkaloid biosynthesis	ko00960	9	39	0.00079123	0.016985096
One carbon pool by folate	ko00670	11	59	0.00144753	0.027417844
Tryptophan metabolism	ko00380	13	81	0.00233318	0.037564152

**Table 3:** **The significantly enriched pathways of down-regulated DEGs**.

**Pathways**	**Id**	**Sample number**	**Background number**	***P*-value**	**Corrected *P*-value**
Pentose and glucuronate interconversions	ko00040	30	100	2.19E-14	7.48E-12
Starch and sucrose metabolism	ko00500	51	331	9.66E-11	1.65E-08
Amino sugar and nucleotide sugar metabolism	ko00520	48	327	1.79E-09	2.04E-07
Phenylalanine metabolism	ko00360	25	120	1.44E-08	1.23E-06
Phenylpropanoid biosynthesis	ko00940	28	153	3.97E-08	2.72E-06
Cysteine and methionine metabolism	ko00270	29	193	1.73E-06	9.86E-05
Glucosinolate biosynthesis	ko00966	9	28	1.60E-05	0.00068345
Inositol phosphate metabolism	ko00562	23	157	2.98E-05	0.00113249
Plant hormone signal transduction	ko04075	57	601	0.000123	0.00350661
Methane metabolism	ko00680	25	203	0.000244	0.0059517
Nitrogen metabolism	ko00910	18	127	0.000319	0.00728052
Glutamatergic synapse	ko04724	13	80	0.000565	0.01206837
Selenocompound metabolism	ko00450	9	45	0.000854	0.01717926
Neuroactive ligand-receptor interaction	ko04080	4	11	0.002514	0.04776096
Mineral absorption	ko04978	7	34	0.002671	0.04808329

### Characterization of Cr stress-responsive genes

Signal sensing and transduction were important participants in response to heavy metal stress. Once entered the plant cell, Cr induces reactive oxygen species (ROS) which were responsible for the activation of MAPK (Mitogen-activated protein kinases) kinase cascade. MAPKs including MAPKKK, MAPKK, and MAPK, were involved in response to various environmental, hormonal and developmental stimuli. Ca/CaM is the signal transduction cascade system, which is associated with to heavy metal stress. In this study, a total of eight DEGs had been identified to be highly homologous to genes encoding MAPKs (*MAPKKK18, MAPKKK19, MAPK3, MAPK16, MAPK17, MAPK18, MAPK19*, and *MAPK20*). In total, 10 DEGs including *CML42, CML44*, and *CML50*, were calcium-dependent protein kinase and calcium-binding protein genes, which were up-regulated under Cr stress in radish roots (Table 4, Table S4).

**Table 4:** **Some critical DEGs responsive to Cr stress in radish roots**.

**Transcript**	**Log_2_FC**	***P*-value**	**FDR**	**Description**
Comp22306_c0_seq1	4.55530704	2.07E-06	2.46E-05	MAPKKK19
Comp47424_c0_seq1	5.31780773	5.79E-05	0.000406339	Putative calcium-binding protein CML44
Comp26037_c0_seq1	4.78683836	4.81E-10	1.92E-08	bZIP transcription factor 60
Comp28465_c1_seq10	34.4390032	4.66E-07	6.86E-06	Ethylene-responsive transcription factor ABR1
Comp19785_c2_seq2	33.6145418	3.57E-05	0.000271789	MYB transcription factor
Comp13434_c0_seq1	3.38377099	4.68E-06	4.93E-05	WRKY DNA-binding protein 15
Comp23733_c0_seq1	−34.180089	9.66E-06	9.02E-05	Peroxidase 17
Comp21825_c0_seq1	−35.687884	2.01E-09	6.63E-08	Peroxidase 21
Comp24452_c0_seq1	4.77201196	6.17E-10	2.40E-08	Peroxidase 34
Comp22278_c0_seq2	−4.7587006	9.50E-05	0.000622163	Peroxidase 49
Comp25683_c0_seq1	−7.5525304	1.36E-16	3.97E-14	Peroxidase 64
Comp26981_c0_seq20	4.34774293	3.70E-07	5.63E-06	Multidrug resistance protein ABC transporter family
Comp27391_c0_seq2	34.0239657	4.28E-06	4.59E-05	ABC transporter-like protein
Comp21544_c0_seq1	7.14134258	3.71E-13	3.81E-11	MATE efflux family protein
Comp50587_c0_seq1	6.15734306	8.76E-07	1.18E-05	Ethylene-responsive transcription factor 9
Comp25404_c0_seq3	38.2011666	1.57E-17	6.33E-15	DNAJ heat shock protein-like protein
Comp25620_c0_seq8	34.0869034	2.94E-06	3.31E-05	HSP20-like chaperone
Comp27769_c0_seq1	3.86061292	1.96E-07	3.28E-06	Glutathione synthetase
Comp25459_c0_seq6	33.4053948	9.64E-05	0.000628082	Cysteine synthase
Comp18928_c2_seq1	8.72713911	4.38E-20	4.06E-17	Glutathione S-transferase
Comp25826_c0_seq2	5.81512529	1.37E-09	4.79E-08	Phytochelatin synthase 1
Comp27638_c0_seq6	−34.141249	1.14E-05	0.000103515	Phytochelatin synthetase-like protein
Comp26972_c1_seq1	−3.2389198	8.27E-06	7.91E-05	Metallothionein type 3
Comp14108_c0_seq2	34.8424264	4.62E-08	9.53E-07	Metallothionein-like protein
Comp20396_c0_seq1	33.8349318	1.17E-05	0.000105136	Cytochrome P450 79b3
Comp20687_c1_seq1	−38.63336	8.55E-18	3.82E-15	Cytochrome P450 79f1
Comp28927_c0_seq1	−6.435114	6.56E-14	8.60E-12	Cytochrome P450 83a1
Comp25273_c0_seq1	7.08334247	2.61E-13	2.84E-11	Cytochrome P450, family 710, subfamily A
Comp29019_c0_seq1	5.02595565	1.09E-10	5.21E-09	CYP83B1
Comp24070_c1_seq2	39.6331444	8.54E-22	1.32E-18	Steroid sulfotransferase 4
Comp14258_c0_seq1	33.8349318	1.17E-05	1.05E-04	Steroid sulfotransferase 3
Comp26378_c0_seq3	3.53992894	1.08E-05	9.96E-05	Fatty acid hydroxylase 1

There were 94 DEGs that were of high homology with different kinds of transcription factors (TFs), such as the WRKY family (i.e., *WRKY1, 6, 13, 26, 28, 33, 46, 48, 50, 70, 71*, and *75*), ethylene-responsive factor (ERF) family (i.e., *ERF008, 012, 018, 070*, 071, *088*, and *098*), myeloblastosis protein (MYB) family (i.e., *MYB1, 4, 28, 29, 44, 46, 51, 58, 73, 95*, and *108*), basic leucine zipper (bZIP) family and dehydration-responsive element-binding protein-type transcription factors (DREB) in this study (Table S4). Among these DEGs, *WRKY6, 26, 28, 33, 50, 71*, and *75, bZIP60, ERF070, 071, 088*, and *098, MYB1, 51, 108* were found to be up-regulated under Cr stress in radish roots (Table 4, Table S4).

High Cr concentration would induce ROS and result in oxidative stress. Accordingly, plants would produce antioxidant enzymes and non-enzymic antioxidants to alleviate ROS damages. In the current, the transcripts encoding peroxidase34, dehydroascorbate reductase (DHAR) and glutathione reductase (GR) were identified to be up-regulated, while peroxidase17, 21, 49, and 64, superoxide dismutase (SOD) and gamma-tocopherol methyltransferase genes were down-regulated in response to Cr stress (Table 4, Table S4).

Metal transporters and chelating compounds play significant roles in coping with HM stress by exclusion, chelation and compartmentalization. A total of 69 DEGs were identified as members of different metal transporter families, which were mainly related to sulfate transporters, heavy metal transport/detoxification domain-containing protein, ATP binding cassette (ABC), ZRT, IRT-like proteins (ZIPs), Natural resistance-associated macrophage proteins (Nramps) families, cation diffusion facilitators (CDFs) and MATE efflux family protein (Table 4, Table S4). Almost all the genes encoding MATE efflux family proteins identified in this study were up-regulated under Cr stress. Four DEGs including three up-regulated and one down-regulated, were found to be homologous with genes encoding metallothioneins (MTs) under Cr stress. In addition, one phytochelatin (PC) related gene (comp25826_c0) was up-regulated in radish roots in the present study (Table 4, Table S4).

Furthermore, Heat shock proteins (HSPs) and Cytochrome P450 (CYP450) genes were also identified in radish roots response to Cr stress. It was reported that HSPs were induced by various kinds of stresses, such as high temperature, heavy metal and drought stress (Dubey et al., 2010). A total of 10 HSP genes are up-regulated in response to Cr stress including two HSP20-like chaperone genes, four heat shock transcription factor (HSF) genes, and two DNAJ HSP-like protein genes, *HSP21* and *HSP22*. Of the four down-regulated *HSPs* three were in the HSP-like family and one *HSP23*. CYP450 genes were involved in a group of secondary metabolite production that helped the plants to resist against various stressful conditions (Balusamy et al., 2013). For example, *CYP79F1, CYP83A1*, and *CYP79B3* involved in glucosinolate biosynthesis were differentially expressed in radish roots under Cr stress. A total of 24 CYP450 genes including 17 up-regulated and seven down-regulated genes were differentially expressed under Cr stress (Table 4, Table S4).

### RT-qPCR validation

**Figure 6:**
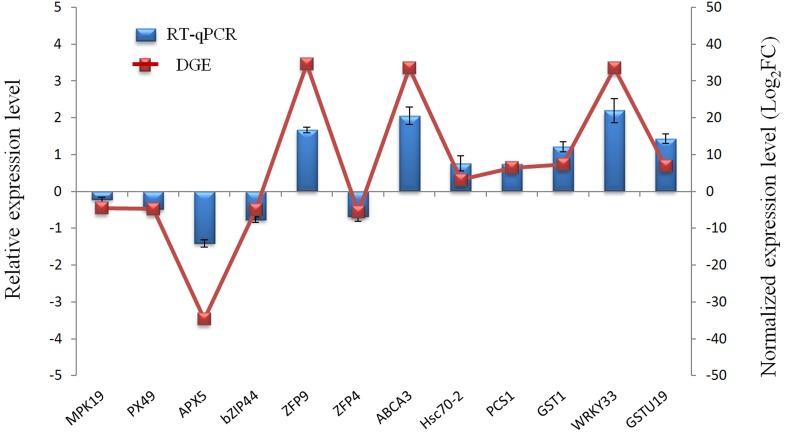
**Validation of the RNA-Seq expression profiles of 12 genes randomly selected from DEGs by RT-qPCR**.

To evaluate the validity of Illumina sequencing and to further confirm the patterns of differential gene expression, a subset of 12 genes highly expressed in the Cr treatment were selected and detected by RT-qPCR analysis with gene-specific primers. These 12 genes were mainly related to defense and detoxification mechanisms including signaling protein kinases (*MPK19*), TFs (*WRKY33, Bzip44*), metal transporters (*ABCA3, ZFP9, ZFP4*), chelate compound biosynthesis (*GSTU19, GST1, PCS1*), antioxidant system (*PX49, APX5*) and HSP (*Hsc70-2*). The expression patterns from RT-qPCR indicated a general agreement with those from the Solexa sequencing results (Figure 6; Table S5).

## Discussion

Chromium (Cr) is considered as a serious environmental pollutant posing a critical concern to human health through food chain (Zayed et al., 1998; Shanker et al., 2005). Exploration of the molecular mechanisms underlying plant response to heavy metal (HM) stress, particularly those facilitate effectively controlling HM uptake, translocation and accumulation, has been a focus work for plant genetic manipulation and genomic research (Wang et al., 2013).

**Figure 7:**
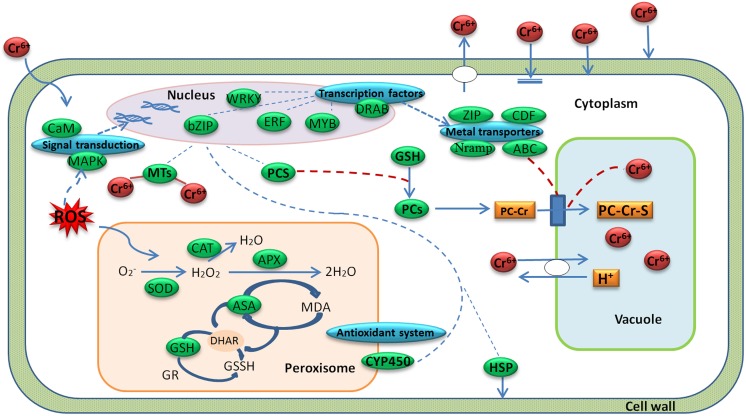
**A regulatory model predicted from the genes expressed differentially during Cr stress in radish roots**. A green box represents a gene enrichment site and corresponding gene or protein name. A blue box represents a gene enrichment site and corresponding gene category.

Radish is an economically important vegetable crop with an edible taproot. The mechanism of radish response to Cr stress has not been clarified at the molecular level before. A global analysis of transcriptome could facilitate the identification of critical gene expression and regulatory mechanisms in plant response to biotic and abiotic stresses such as cold (Shan et al., 2013), salinity (Dang et al., 2013) and HM stress (Yamaguchi et al., 2010; Yu et al., 2012; Li et al., 2014). In the present study, with a transcriptome profiling of radish root, some critical DEGs responsive to Cr stress were discovered, and the hypothesis model of Cr stress-responsive gene regulatory network in radish roots was proposed (Figure 7).

### The role of antioxidant system in plant response to Cr stress

Plants exposed to toxic heavy metal (HM) can generate reactive oxygen species (ROS), resulting oxidative stress (Panda and Choudhury, 2005). Oxidative stress can lead to lipid peroxidation in plants and disrupt the cellular functions, resulting in inhibition of plant growth and development (Yadav, 2010). To fight against oxidative stress, plants have evolved a complex antioxidant system for elimination of ROS to protect the cell from damage. Actually, the defense system including enzymatic and non-enzymatic antioxidants was considered as an important mechanism of metal detoxification and tolerance in plants. SOD is the first line of defense against ROS-mediated damage and could scavenge toxic O^−^_2_ in plant cells (Zeng et al., 2012). POD, CAT, and the ascorbate–glutathione cycle (APX and GR) play crucial roles in scavenging H_2_O_2_. Cr has the redox behavior which exceeds that of other metals including Co, Fe, Zn, and Ni (Panda and Choudhury, 2005). In the current study, some functional genes encoding peroxidase17, 21, 34, 49, and 64, SOD, DHAR, tocopherol and GR, were identified in the radish root exposed to Cr (Table 4, Table S4). It was interesting that almost all the DEGs related to peroxidase were down-regulated under Cr stress, which might result from the cellular injury including bio-membrane lipid peroxidation under Cr stress.

Glutathione is also an important antioxidant involved in cellular defense against toxicants. GSH is a redox buffer protecting the cytosol and other parts of cells against ROS which are induced by biotic and abiotic stresses (Zitka et al., 2013). In this study, one cysteine synthase (comp25459_c0), two glutathione synthetase (GSS) and 17 out of 19 glutathione S-transferase (GSTs), were up-regulated in radish roots following Cr stress (Table 4, Table S4). These findings provided further evidence for the role of GSH in antioxidant metabolism. Furthermore, GSH, serving as a precursor, could participate in the synthesis of phytochelatins (PCs). Therefore, it could be concluded that antioxidant system plays a fundamental role in plant cellular detoxification.

### Signal transduction and TFs in plant response to Cr stress

When encountered with extra-cellular stimuli or to prevent HM entrance from susceptible sites into the protoplast, the cell walls could activate a variety of specific stress-responsive signaling proteins, such as MAPKs and calcium-binding related proteins. It was reported that MAPKs were important mediators in signal transmission, connecting the perception of external stimuli to cellular responses (Sanchita Dhawan and Sharma, 2014). The calmodulin (CaM) system could regulate HM ion transport, gene expression, and stress tolerance (DalCorso et al., 2010). In this study, *MAPK18, MAPK20*, and other calcium-binding related protein genes were expressed differentially in the radish root under Cr exposure. These genes were also found to be expressed differentially in radish under Pb stress, indicating that MAPKs and calcium-binding related proteins were involved in signal transduction in plants under HM stress (Wang et al., 2013).

Once phosphorylated, MAPKs enter into the nucleus through the phosphorylation of TFs to regulate gene expression. A series of TFs including bZIP, WRKY, ERF, and MYB have been shown to play important roles in regulating the expression of specific stress-related genes (Thapa et al., 2012). In this study, different kinds of TFs including up-regulated *WRKY, ERF, MYB* and *bZIP60* were identified in radish response to Cr stress (Table 4, Table S4). It could be concluded that the plants would activate a series of TFs to regulate corresponding transcriptional processes to alleviate the phytotoxicity of HMs.

### Transporter and chelate compound in plant response to Cr stress

It was reported that a large number of transporter families including ABC, Nramps, ZIPs, CDFs, and MATE efflux family proteins involved in HM uptake, transport, distribution and plant tolerance to HM stress (Dubey et al., 2010; Lan et al., 2013). A large number of transporters including sulfate transpoters, MATE-efflux family proteins, ABC transporter family proteins, heavy metal-associated domain containing proteins, were differentially expressed after challenge with Cr in the rice root (Dubey et al., 2010). Wang et al. (2013) reported that in radish response to Pb stress, the main differentially expressed metal transpoters were ABC, Nramps, ZIPs, and CDFs, which were also identified in the present study (Table 4, Table S4). These results indicate that there would be similar mechanisms underlying the uptake, transport and detoxification of HMs among different plant species.

It was found that PCs and MTs could chelate metal ions to form metal-chelate compounds and sequester them in the vacuole (Sharma and Chakraverty, 2013). MTs are low-molecular-weight cysteine-rich metal binding peptides. Under HM stresses, different MT types have been reported in plants including oil palm (Abdullah et al., 2002), poplar (Kohler et al., 2004), and radish (Wang et al., 2013). In the present study, the genes encoding MT3 and MT-like proteins were also found under Cr stress in the radish root. Moreover, two phytochelatins (PCs) related genes were also identified (Table 4, Table S4). It was reported that PCs have a high affinity in binding HMs, and PC–HM complexes are then transported into the vacuole, leading to inactivation of HMs in the plant cells (Zeng et al., 2012).

### CYP450s and HSPs may be involved in plant response to Cr stress

CYP450s play crucial roles in biosynthesis of a variety of endogenous lipophilic compounds such as fatty acids, sterols, phenylpropanoids, terpenoids, and phytoalexins brassinolides, which could enhance the plant tolerance under stresses (Narusaka et al., 2004; Vázquez et al., 2013; Fariduddin et al., 2014). It was reported that CYP450s were induced by abiotic stress, such as As (Chakrabarty et al., 2009) in rice, Pb (Liu et al., 2009) in *Arabidopsis*, Cr (Dubey et al., 2010) in rice and dehydration stress in cabbage (Yu et al., 2012), and biotic stress including insecticide stress in ladybirds (Zhang et al., 2012) and bacterial infection in tobacco (Daurelio et al., 2011). The present results showed that a total of 24 CYP450 genes were differentially expressed in the radish root under Cr stress. It was interesting that biotic and heavy metal stress response in plants may have a similar mechanism (Rai and Mehrotra, 2008).

Furthermore, HSPs, which are molecular chaperones, have the function of repairing proteins under stress conditions. Hsp90-1 was found to be highly accumulated in tomato under Cr and As stress (Goupila et al., 2009). Three up-regulated and five down-regulated HSP genes were identified under Cr stress in rice (Dubey et al., 2010). In this study, a similar result was obtained and a total of 14 HSP genes were identified in the radish root under Cr stress. It could be inferred that HSPs play vital roles in enhancing the plant tolerance to Cr stress.

### Cr stress-response regulatory networks in radish

Plants have developed a variety of complex physiological and genetic mechanisms to cope with HM toxicity. In general, there are several strategies to defense and regulate HM stress including immobilization, exclusion, chelation and compartmentalization (Hall, 2002; Sharma and Chakraverty, 2013). Based on the DEGs enrichments with the technique, Cr stress-response regulatory network was put forward in radish roots (Figure 7). As shown in Figure 7, when a plant cell was exposed to Cr, oxidative stress would be induced. The oxidative stress could generate lipid peroxidation and cause severe damages to bio-membranes in plant cells (Panda and Choudhury, 2005). In order to decrease excess levels of oxidative damage, several metabolites and enzymes including GPX, SOD, APX, and CAT were activated. After sensing the signal, the plant cells activated some Cr stress responsive signaling molecules such as MAPKs and CMLs, which then guided TFs (WRKY, bZIP, ERF, MYB families) in the nucleus to regulate the expression of some functional genes. These genes may encode transporters (ABC, ZIP, Nramps, and CDFs) and chelating compounds (MTs and PCs), which would alleviate Cr toxicity via diverse mechanisms in plant cells (Figure 7; Table 4; Table S4).

## Conclusion

The comprehensive transcriptome-based characterization of Cr responsive differentially expressed genes (DEGs) was firstly conducted in radish roots. This result revealed that the expression of 1424 unigenes was up-regulated, and 1561 unigenes were down-regulated in radish roots under Cr stress. Most of the DEGs were relative to antioxidant system, signal transduction and TFs, transporters and chelate compounds biosynthesis. In addition, a model of Cr stress-response regulatory network in the radish root was proposed. These results would provide useful information for dissecting the molecular genetic mechanism underlying Cr uptake, sequestration, translocation and detoxification in root vegetable crops.

## Accession code

The RNA SEQ raw data have been deposited in NCBI Sequence Read Archive (SRA, http://www.ncbi.nlm.nih.gov/Traces/sra) with accession numbers: SRX256970 (CK) and SRX862647 (Cr600).

### Conflict of interest statement

The authors declare that the research was conducted in the absence of any commercial or financial relationships that could be construed as a potential conflict of interest.
