# Supplementary material for: Transcriptome-based gene profiling provides novel insights into the characteristics of radish root response to Cr stress with next-generation sequencing
Source: Front Plant Sci. 2015 Mar 31;6:202. doi: 10.3389/fpls.2015.00202 (PMC4379753; doi:10.3389/fpls.2015.00202)
Supplement: Supplementary file 5 [file Table5.DOCX]

**Table S5** Primers used for the qRT-PCR.

| Unigene (name) | Primer sequence | | | Amplicon size (bp) |
| --- | --- | --- | --- | --- |
|  | Forward (5’-3’) | | Reverse (5’-3’) |  |
| GST1 | AACCAATCCGTGAATCTC | GGAGTTCCAAGCAAGTAT | | 147 |
| MPK19 | TAGTGCTATTGGTCATCTG | TCTCTGGAGGTGGTATG | | 89 |
| Px49 | GGAGGATAGTGAGTGAGAA | AGTGAGAACAGAGGAGTC | | 161 |
| APX5 | CATCCACGAGTCTCTTAC | TCCTTCATCTGCTGTCT | | 123 |
| DWRKY33 | TCATCATCTTCTTCTTCTTCA | GAGGTAGTATTATCATCTTGTTG | | 134 |
| bzip44 | CTCAGGTCGCTCATCTA | CAGTCTCCATCTCAAAGG | | 192 |
| RZFP9 | GCTTCATTGTTCGTGTC | CTCTTCTTGCTCAGTAGG | | 160 |
| ZFP4 | CCAACACAAGAGACGAT | TCACCTAAATCACCATCAA | | 170 |
| ABCA3 | TCCTCACGACTCATTCTA | CCAACATCTCCACATCTT | | 177 |
| Hsc70 -2 | GGAGAAGAGAAGCAGTT | TGAGAGTCGTTGAAGTAG | | 137 |
| BjPCS1 | TTCAAGTATCCTCCTCATT | CAATCCTGGTTCTCTGT | | 126 |
| GSTU19 | TATCTCAATGAACTCCTTCTT | CCTATCCTCCCTTCTGAT | | 147 |
